# Association of γδ T Cell Compartment Size to Disease Activity and Response to Therapy in SLE

**DOI:** 10.1371/journal.pone.0157772

**Published:** 2016-06-22

**Authors:** Hongshuang Ma, Yi Yuan, Ling Zhao, Zhuang Ye, Jiandong Xu, Man Li, Zhenyu Jiang, Yanfang Jiang

**Affiliations:** 1 Department of Rheumatology, the First Hospital, Jilin University, Changchun, 130021, China; 2 Genetic Diagnosis Center, the First Hospital of Jilin University, Changchun, 130021, China; 3 Key Laboratory for Zoonosis Research, Ministry of Education, the First Hospital, Jilin University, Changchun, 130032, China; 4 Jiangsu Co-innovation Center for Prevention and Control of Important Animal Infectious Diseases and Zoonoses, Yangzhou, 225009, China; 5 Westbury Christian School, 10420 Hillcroft, Houston, TX, 77096, United States of America; Instituto Nacional de Ciencias Medicas y Nutricion Salvador Zubiran, MEXICO

## Abstract

**Objective:**

Although γδT cells are widely recognized as pivotal elements in immune-mediated diseases, their role in the pathogenesis of SLE and therapeutic outcome remains under explored. The current study aims to characterize the γδT cell compartment in SLE and correlate its status to disease severity and response to therapy.

**Methods:**

Human peripheral blood-derived γδ T cells were isolated from 14 healthy volunteers and 22 SLE patients (before and after 4 and 12 weeks following the onset of glucocorticoids (GC), mycophenolatemofetil (MMF) orhydroxychloroquine (HCQ) treatment). The γδ T cells were characterized using flow cytometry. In addition, serum concentration of IFN-γ, TNF-α, IL-2, IL-4, IL-6, IL-10 and IL-17A was determined by cytometric bead array (CBA).

**Results:**

The SLEDAI scores dropped significantly following therapy in a subset of patients (responders–R) but not in some (non- responders–NR). Peripheral blood γδ T cells in general, and γ9^+^δ T cells and TNF-α/IL-17-secreting CD4^-^CD8^-^γδ T cell subsets in particular, were decreased in SLE compared to healthy controls. The numbers of the γδ T cell subsets reached levels similar to those of healthy controls following therapy in R but not in NR. Serum IL-6, IL-10 and IL-17 but not IFN-γ and TNF-α were significantly increased in SLE compared to the healthy controls and exhibited differential changes following therapy. In addition, inverse correlation was observed between SLEDAI scores and γδ T cell compartments, especially with TNF-α^+^γδT cells, TNF-α^+^γ9+δT cells and IL17^+^CD4^-^CD8^-^γδT cells subsets. Differential correlation patterns were also observed between serum cytokine levels and various γδ T cell compartments.

**Conclusions:**

A strong association exists between γδ T cell compartments and SLE pathogenesis, disease severity and response to therapy.

## Introduction

SLE is an autoimmune disease which is characterized by the presence of auto-antibodies against nuclear antigens, immune complex formation, localized and generalized inflammation, followed by progressive injury to the affected organ and resulting in its loss of function [1]. It is now well-established that its pathogenesis involves the idiopathic activation of self-reactive T and B cells that subsequently play important roles in tissue damage. Within the set of these immune cells, γδ T cells are potential mediators of the production of pro-inflammatory cytokines and pathogenic auto-antibodies, and possibly involved in the onset of this autoimmune disease [2].

T cells with its antigen receptor (TCR) bearing α and β subunits (αβT cells) constitute the vast majority of human T lymphocytes, and those bearing γ and δ subunits (γδT cells) are relatively less abundant. This latter type of T lymphocytes, the so-called [3] γδT cells are present in peripheral blood, skin and mucosal surfaces, spleen and lymph nodes and facilitates interaction between innate and cell-mediated immune [4]. The major functions of γδT cells include perforin-mediated killing of tumor cells [5], antigen presentation [6–7], cytokine production [8] and pathogen phagocytosis [9]. The γδT cells exist mainly as either δ1 cells or γ9δ2 cells. And the latter is predominantly present in the circulation and accounts for 0.5–5% of T cells in the peripheral blood where they appear to assist host defense in an apparently TCR-independent fashion [5]. In contrast, the δ1T cells are the main γδT cell component of the skin and mucosal epithelia, where they account for 10% and 40% of all T cells respectively [10–11]. δ1T cells are relatively underexplored, but they have been suggested to possess regulatory function [12]. The potential regulatory cells in skin and mucosal tissues which are frequently affected by SLE raise obvious questions as to their potential functionality in the initiation and/or progression of SLE.

Indeed, previous studies have reported about γδT cells in SLE, however, the exact role for these cells has not been clarified [13–15]. Thus further studies are required to elucidate the contribution of γδT cells in general, and as well as the potential role of specific subsets of γδT cells in the progression of disease and their influence on responses to therapy in particular. Currently, SLE patients are stratified for therapy based on disease severity, extent of immune cell organ infiltration, economic situation and so on. More advanced cases require treatment with glucocorticoids (GC) and immunomodulators like mycophenolate mofetil (MMF) or hydroxychloroquine (HCQ) [16]. The current study investigates the relationship between the status of peripheral blood γδT cell compartment and disease severity. In addition, the study also characterized the changes in the different γδT cells subsets in the peripheral blood of SLE patients following GC, HCQ and MMF therapy and after treatment such changes in γδ T cell properties returned to normal values. The results support an important negative role for γδT cell compartment in the pathogenesis of SLE.

## Results

### Patient characteristics and clinical response to therapy

A total of 22 SLE patients and 14 healthy controls were recruited to investigate the γδT cell compartment in SLE and its relation to the dynamic evolution of the disease and therapeutic strategy. Demographic variables, disease characteristics and clinical course are provided in Table 1. Significantly high SLEDAI scores in SLE patients prior to treatment reduced markedly following GC, MMF and HOQ therapy. Similarly, the concentrations of serum C3 and C4 significantly increased and the levels of anti-dsDNA antibodies decreased. Further analysis revealed a dichotomy in clinical response to therapy in SLE patients. Patients who exhibited favorable clinical response to therapy (SLEDAI ≤ 6.0) were categorized as responders (R) and those who failed to reach the expected clinical response (SLEDAI > 6.0) were grouped as non-responders (NR). After 12 weeks’ treatment, there were only 3 patients whose SLEDAI > 6.0 and were thus classified as non-responder (NR), while others were classified as responders (R). The R group consists of 19 patients before treatment and 13 patients in four weeks and 14 patients in twelve weeks following therapy. The SLEDAI score in R group was found to be significantly lower than that of the non- responders (NR) group following therapy (Table 2). The non-responders (NR) failed to show significant changes in other clinical parameters compared to that of zero week controls. We concluded that our cohort provides us with an opportunity to link the γδT cell compartment to disease activity and study the dynamics evolution of SLE in a variety of clinical responses to therapy.

**Table 1:** Demographic and clinical parameters of study participants.

	SLE patients			Healthy controls
	0 week	4 weeks	12 weeks	
Number of patients	n = 22	n = 16	n = 17	n = 14
Age in years	39 (23–58)			41 (24–57)
SLEDAI	14 (6–23)	6(1–18)*	3(0–8)*	-
Positive Anti-dsDNA	17 (77.3%)	8 (50%)*	5 (29.4%)*	-
Positive Anti-Sm	6 (27.7%)	5 (31.25%)	2 (11.76%)	-
C3 (mg/L)	0.50 (0.05–1.16)	0.98 (0.59–1.25)*	1.17 (0.73–1.56)*	-
C4 (mg/L)	0.10 (0.01–0.49)	0.14 (0.05–0.25)*	0.18 (0.06–0.34)*	-
CRP (mg/L)	5.44 (0.86–24.4)	2.02 (0.16–11.2)	3.41 (0.16–9.31)	-
ESR(mm/H)	52.4(3–111)	18.5(3–91)	24(4–61)	-
WBC (x10^9^/L)	3.66 (0.65–7.05)	9.46 (3.42–15.4)*	9.81 (3.14–18.93)*	6.80 (4.44–10.3)*
PLT(x10^9^/L)	132 (26–293)	189 (95–315)*	227 (138–353)*	212 (128–295)*
LY (x10^9^/L)	0.78 (0.31–2.18)	1.69 (0.7–2.71)*	2.59 (1.1–6.75)*	2.1 (1.49–3.51)*

Data shown are median (range) for each group of subjects, except for those specified otherwise. SLEDAI: systemic lupus erythematosus (SLE) disease activity index; CRP: C-reactive protein; C3 and C4: complements 3 and 4; WBC: white blood cells; PLT: blood platelet; LY: lymphocytes; 0 week: baseline or before therapy; 4 weeks and 12 weeks: 4 and 12 weeks after therapies

*: *P*<0.05 vs. baseline values.

(–) indicates levels undetected.

**Table 2:** Stratification analysis of the clinical measures in patients following regular therapy for SLE.

	0 week		4 weeks		12 weeks	
	R(19)	NR(3)	R(13)	NR (3)	R (14)	NR (3)
SLEDAI	13 (6–23)	14 (6–19)	5 (1–11)*	10 (4–18)	2.5 (0–4)*	8 (8)
Positive Anti-dsDNA	13	3	6	2	7	0
Positive Anti-Sm	6	0	5	0	2	0
C3	0.48(0.05–1.16)	0.69(0.53–0.85)	0.97(0.59–1.25) *	0.92(0.68–1.15)	1.18(0.73–1.56) *	1.11(1.07–1.13)
C4	0.07(0.01–0.16)	0.30(0.12–0.49)	0.14(0.05–0.25)*	0.12(0.12–0.13)	0.18(0.06–0.34) *	0.16(0.14–0.21)
CRP	5.99(0.62–24.4)	1.57(0.86–3.0)	2.42(0.17–11.2)	3.77(0.16–10.3)	5.69(0.16–38.1)	3.3(0.3–9.31)
ESR	52.5(3–111)	46.7(31–69)	18(3–91)	10(5–15)	25.3(4–61)	5.67(5–6)

Data shown are median (range) of each group of subjects.

*: *P*<0.05 vs baseline values.

The patients having an adequate clinical response to therapy (i.e. a SLEDAI <6.0) were defined as well responders (WR) and the remaining patients were grouped as poor responders (PR).

### SLE is characterized by reduced number of peripheral blood γδT cells and subsets

**Fig 1:**
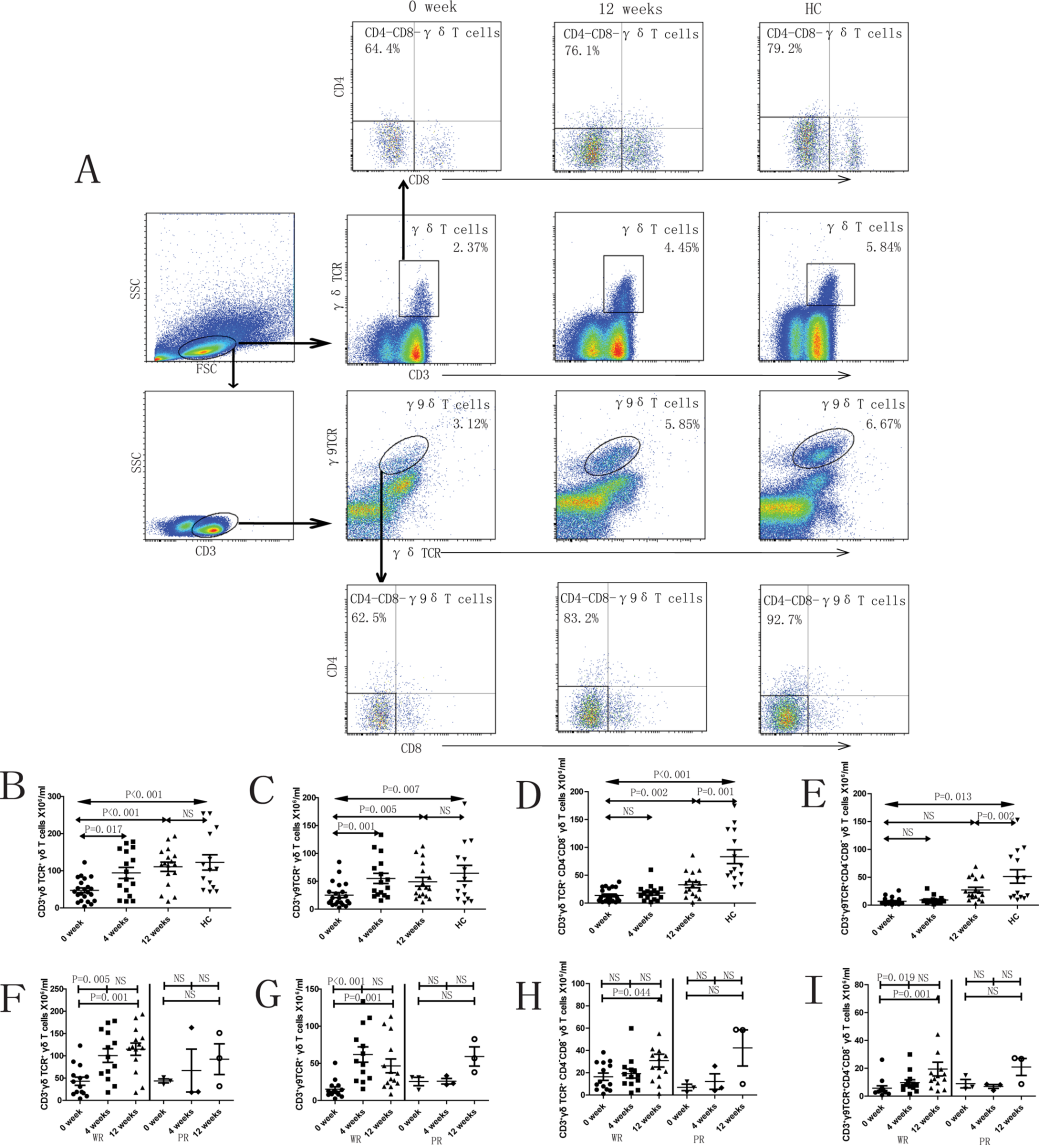
Peripheral blood γδ T cells and subsets in SLE patients. A. The panels in this section show the gating strategy employed for the analysis of γδ T cells and subsets. Peripheral venous blood derived leukocytes were stained with different fluorescent antibodies and after lysis of red blood cells, the remaining cells were gated on living lymphocytes, then gated on CD3^+^γδTCR^+^ or CD3^+^γδTCR^+^γ9TCR^+^ cells, and then further gated on CD4^-^CD8^-^ cells, respectively. B—I. Flow cytometry results are represented as the scatter dot plots of γδ T cells and subsets in healthy controls (HC) or in SLE patients before and after therapy (treatment time is indicated). SLE patients are further grouped as responders (R) and non-responders (NR) based on response to therapy.

Significantly lower number of γδ T cells (p<0.001) and subsets– γ9^+^δ T cells (p = 0.007) was observed in SLE patients (0 week, prior to treatment) compared to healthy controls (Fig 1B and 1C). Similar trends were found in the numbers of CD4^-^CD8^-^γδT cells and CD4^-^CD8^-^γ9^+^δT cells (Fig 1D and 1E). The numbers of γδT cells and γ9^+^δT cells in SLE patients increased significantly at 4 and 12 weeks following onset of treatment (Fig 1B and 1C). It was observed that in R group the numbers of γδT cells, γ9^+^δT cells and CD4^-^CD8^-^γ9^+^δT cells increased after 4 and 12 weeks of treatment (Fig 1F, 1G and 1I). In contrast, the number of these cells in the NR group remained unchanged after the onset of treatment (Fig 1F, 1G and 1I).

**Fig 2:**
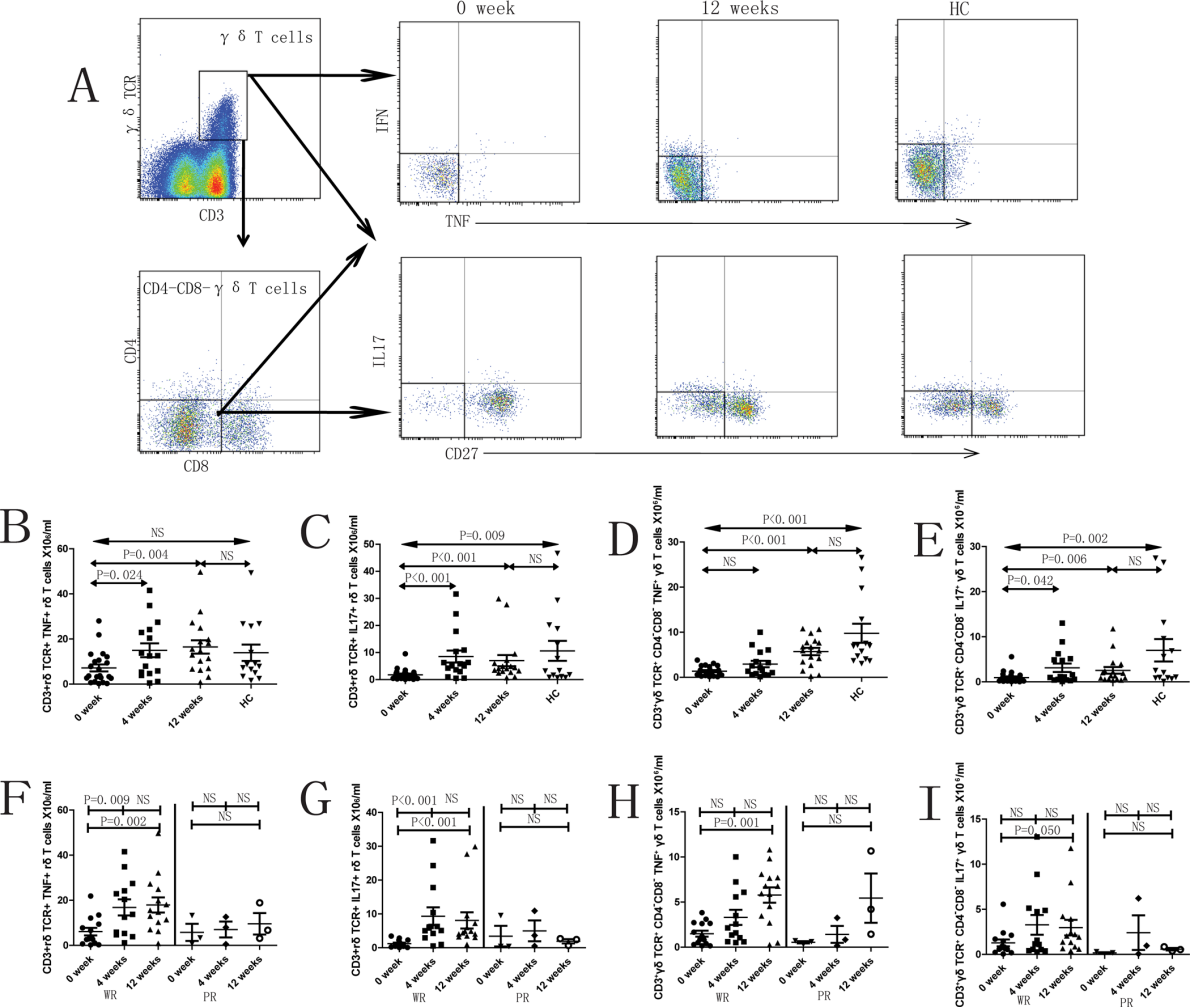
Characterization of different subsets of CD3^+^γδTCR^+^ cells in SLE patients. A. The panels in this section show the gating strategy employed for the analysis of γδ T cells and subsets. Peripheral venous blood derived leukocytes were stained with different fluorescent antibodies and after lysis of red blood cells, the remaining cells were gated on living lymphocytes and further gated on CD3^+^γδTCR^+^ cells and CD3^+^γδTCR^+^CD4^-^CD8^-^ cells, and then further gated on IFN-γ^+^, TNF-α^+^, IL17^+^ and CD27^+^ cells, respectively. The frequencies of different subsets of γδT cells were analyzed by flow cytometry. B—I. Flow cytometry results represented as the scatter dot plots of γδ T cells and subsets in healthy controls (HC) or in SLE patients before and after therapy (treatment time is indicated). SLE patients are further grouped as responders (R) and non-responders (NR) based on response to therapy.

**Fig 3:**
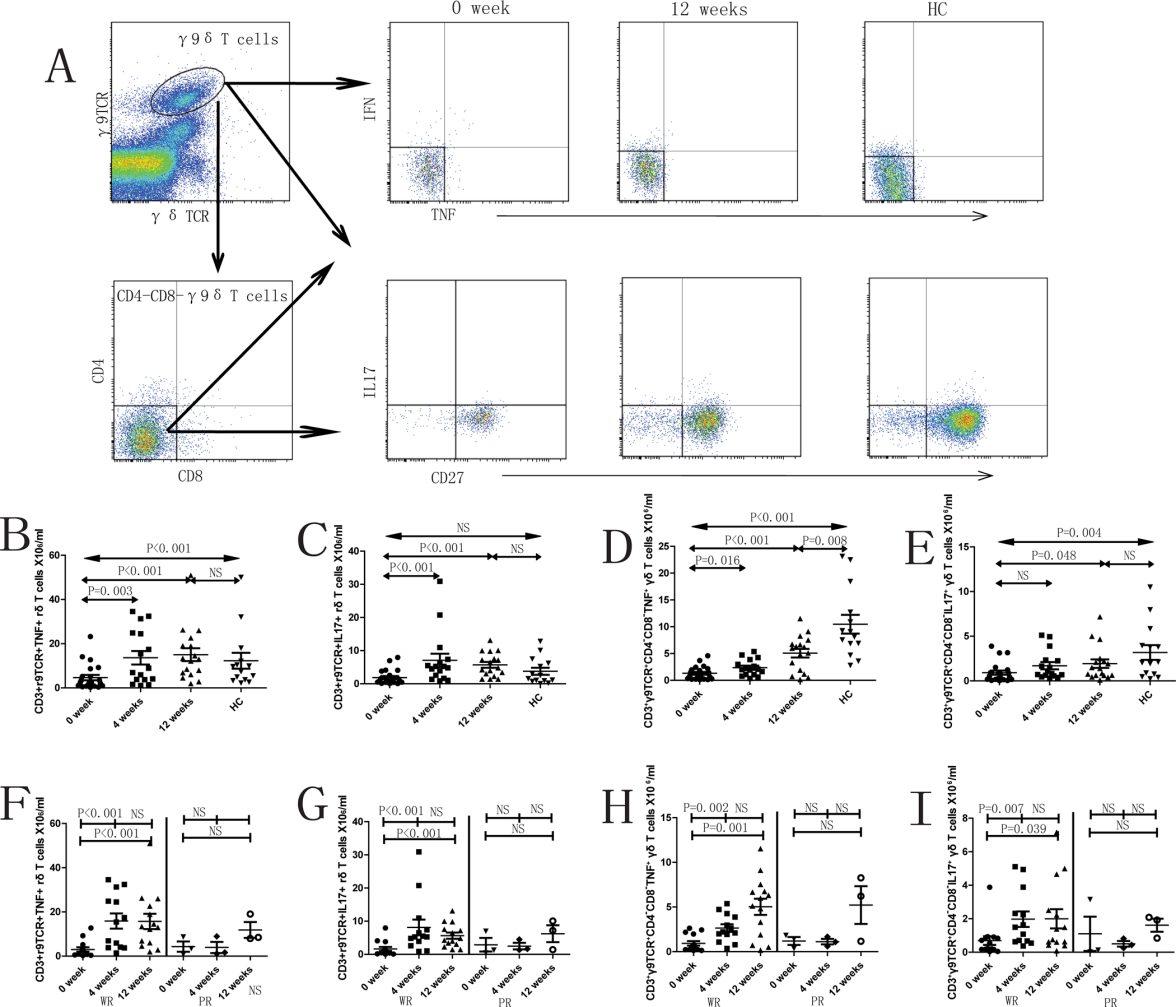
Characterization of different subsets of CD3^+^γδTCR^+^γ9TCR^+^ cells in SLE patients. A. The panels in this section show the gating strategy employed for the analysis of γδ T cells and subsets. Peripheral venous blood derived leukocytes were stained with different fluorescent antibodies and after lysis of red blood cells, the remaining cells were gated on living lymphocytes and further gated on CD3^+^γδTCR^+^γ9TCR^+^ cells and CD3^+^γδTCR^+^γ9TCR^+^CD4^-^CD8^-^ cells, and then further gated on IFN-γ^+^, TNF-α^+^, IL17^+^ and CD27^+^ cells, respectively. The frequency of different subsets of γ9δT cells were analyzed by flow cytometry. B—I. Flow cytometry results represented as the scatter dot plots of γδ T cells and subsets in healthy controls (HC) or in SLE patients before and after therapy (treatment time is indicated). SLE patients are further grouped as responders (R) and non-responders (NR) based on response to therapy.

In addition, the compartment sizes of IL17^+^ γδT cells, TNF-α^+^CD4^-^CD8^-^γδT cells and IL17^+^CD4^-^CD8^-^γδT cells in peripheral blood were significantly lower in SLE patients (0 week, prior to treatment) compared to healthy controls (Fig 2C–2E). The numbers of these subsets of cells as well as TNF-α^+^γδT cells increased significantly following treatment in the R group. However, this effect was not observed in the NR group (Fig 2F–2I). Similarly, the numbers of TNF-α^+^γ9^+^δT cells, TNF-α^+^CD4^-^CD8^-^ γ9^+^δT cells and IL17^+^CD4^-^CD8^-^ γ9^+^δT cells were significantly lower in SLE patients (0 week, prior to treatment) compared to healthy controls (Fig 3B, 3D and 3E). The numbers of this cell subsets as well as IL17^+^γ9^+^δT cells reached similar numbers to those of healthy controls after 4 and/or 12 weeks treatment in R group but not in the NR group (Fig 3F–3I). The γδT cell subsets secreting IFN-γ had similar trends (data not shown). Thus, γδT cell and its subset numbers were inversely proportional to disease severity and were observed to improve following therapy with reduced disease severity.

### Serum cytokines profile in SLE patients

**Fig 4:**
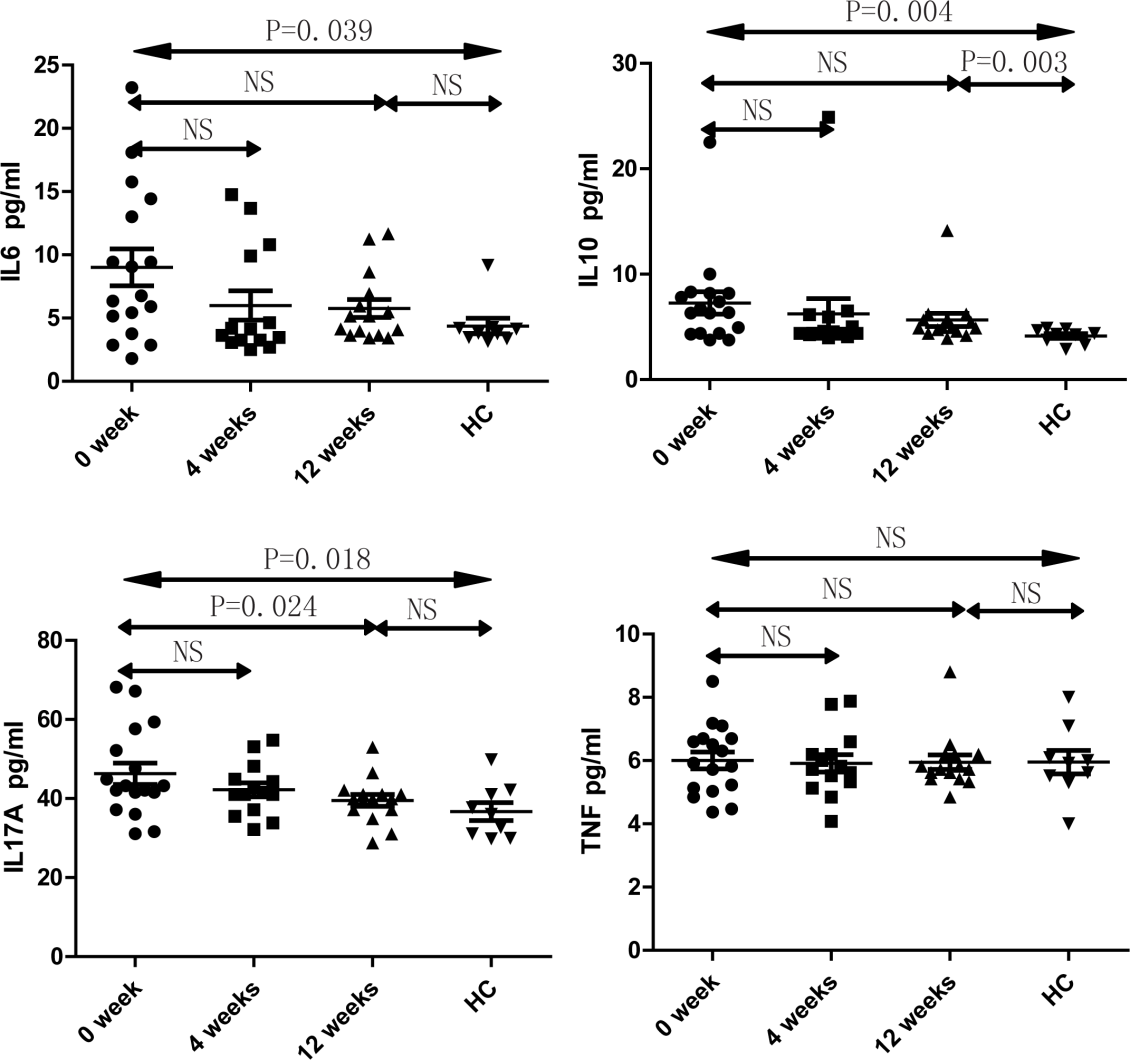
Cytokines profile in SLE patients The levels of serum IL-2, IL-4, IL-6, IL-10, IL-17A, IFN-γ and TNF-α in study participants was analyzed by CBA before and at 4 weeks and 12 weeks after initiation of treatment.

In order to obtain an insight into the observed changes in systemic changes in immunological status, we examined the levels of serum cytokines including IL-2, IL-4, IL-6, IL-10, IL-17A, IFN-γ and TNF-α in study subjects using CBA. We observed that the concentrations of serum IL-17A, IL-6 and IL-10 are significantly higher in the SLE patients (0 week, prior to treatment) compared to that of healthy controls (Fig 4). The level of serum IL-17A was found to be significantly reduced following 12 weeks of treatment, without significant changes in the levels of IL-2, IL-4, IFN-γ and TNF-α in SLE patients under therapy (Fig 4). Furthermore, the association between the levels of these cytokines and various clinical indicators was also performed.

### Correlations between the peripheral blood γδT cell subset composition and clinical indicators in SLE patients

**Fig 5:**
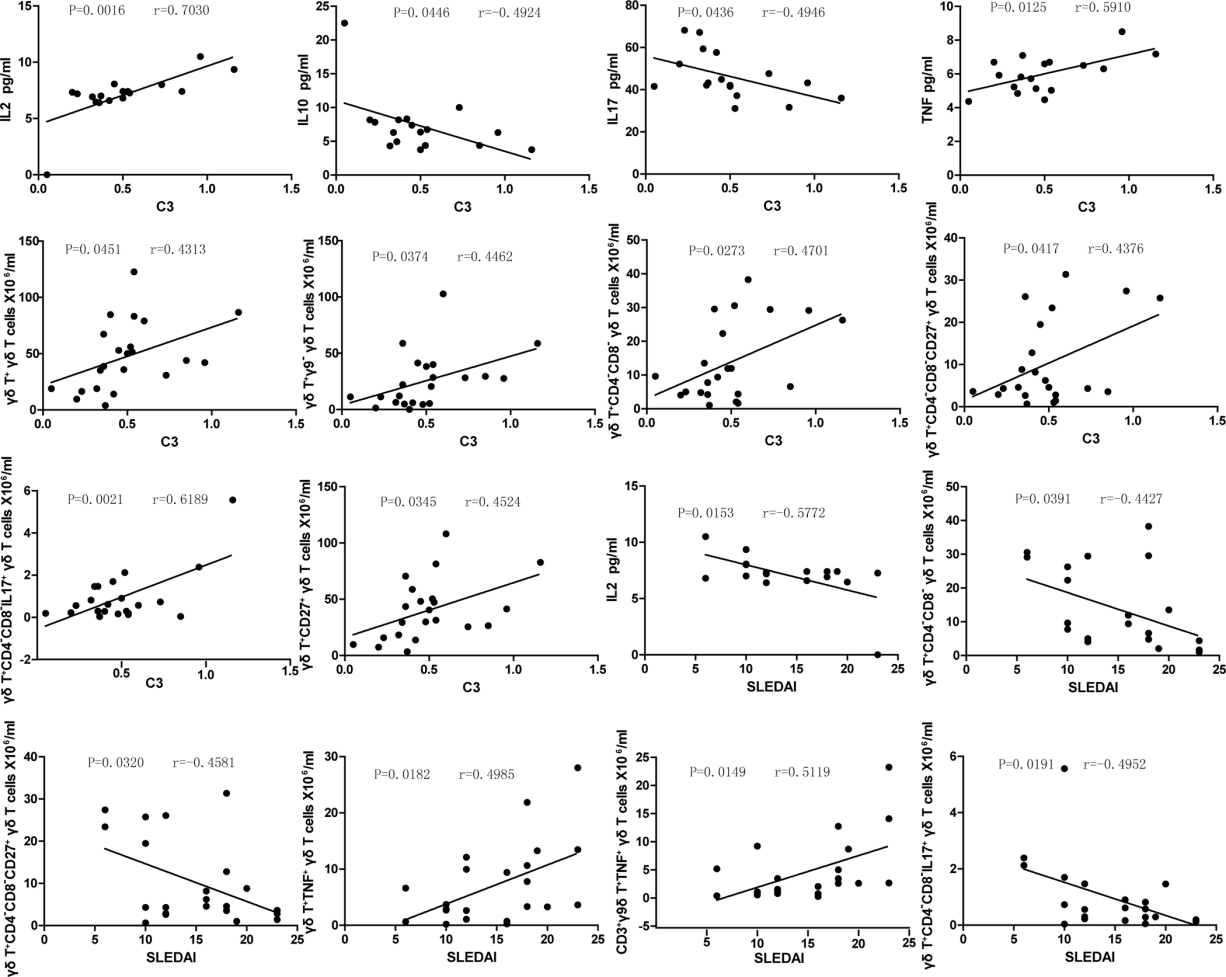
Correlations between γδT cell subsets size and the clinical indicators. The levels of the serum C3 and SLEDAI scores were correlated to various serum cytokines and to *pan* γδT cell numbers and to γδT cell subsets in SLE patients. There is no significant correlation among other parameters tested (data not shown), see text for details.

We analyzed the potential relationship between the different subsets of γδT cells and relevant clinical indicators such as SLEDAI scores, serum C3 levels and serum C4 levels. It was observed that the serum C3 and SLEDAI scores correlated with several serum cytokine levels and with size of γδT cell compartments (Fig 5). As shown in Fig 5, IL-2, TNF-α and the numbers of peripheral blood γδT cells, γ9^-^δT cells, CD4^-^CD8^-^γδT cells, CD4^-^CD8^-^CD27^+^γδT cells, IL17^+^CD4^-^CD8^-^γδT cells and CD27^+^γδT cells showed positive correlation with serum C3 level in SLE patients. Whereas, IL-10 and IL-17 exhibited negative correlation with serum C3 level in SLE patients (Fig 5). Additionally, the size of TNF-α^+^γδT cells and TNF-α^+^9γ^+^δT cell compartments showed positive correlation with SLEDAI scores while serum IL-2 levels and CD4^-^CD8^-^γδT cells, CD4^-^CD8^-^CD27^+^γδT cells and IL17^+^CD4^-^CD8^-^γδT cell numbers correlated negatively with SLEDAI scores (Fig 5). The correlation pattern observed further highlights the potential role of γδT cell compartments in the pathogenesis of SLE.

### Correlations between the different γδT cells subsets in the SLE patients

**Fig 6:**
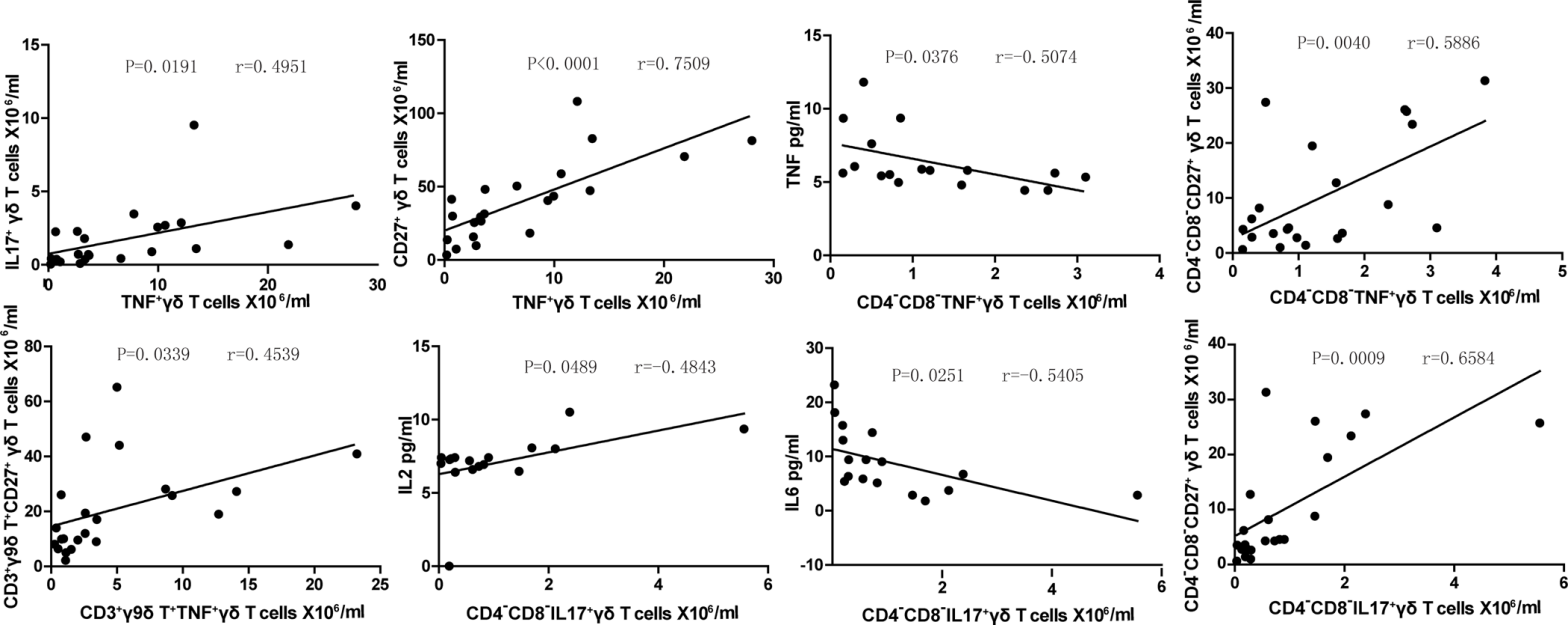
Correlations between the different subsets of γδT cells. The levels of the numbers of different subsets of γδT cell were correlated in SLE patients. Apart from those shown no statically significant correlations were detected.

The potential relationships between the sizes of the different γδT cell subsets in SLE patients were also analyzed. We observed that the number of IL17^+^γδT cells and CD27^+^γδT cells showed positive correlation with the number of TNF-α^+^γδT cells (Fig 6). The number of CD4^-^CD8^-^CD27^+^γδT cells correlates well with the number of the TNF-α^+^CD4^-^CD8^-^γδT cells, but interestingly serum TNF-α levels showed a negative correlation with the number of TNF-α^+^CD4^-^CD8^-^γδT cells (Fig 6). CD27^+^γ9^+^δT cell numbers correlated positively with the number of TNF-α^+^γ9^+^δT cells, and serum IL-2 and CD4^-^CD8^-^CD27^+^γδT cell compartment size showed a positive correlation with the number of the IL17^+^CD4^-^CD8^-^γδT cells, whereas serum IL-6 levels exhibited negative correlation with IL17^+^CD4^-^CD8^-^γδT cells (Fig 6). The multiple correlations observed between different γδT cell subsets and serum cytokine levels further suggest the contributions of the different γδT cell subsets in the pathogenesis of SLE.

## Discussion

γδT cells are an important group of T lymphocytes that play a major role in host immunity providing protection against infectious disease and cancer. Recently, γδT cells have also been implicated in the pathogenesis of autoimmune diseases including SLE [2, 13]. Nevertheless, a detailed knowledge regarding its role in SLE is lacking. An earlier study has shown that the γδT cell compartment was larger in new-onset SLE patients compared to healthy controls and its size reverted back to normal levels following therapy [15]. However, a recent study has reported otherwise, by showing that the number of γδT cells is decreased in SLE patients [13]. Although all these studies indicate a link between the γδT cell compartment and SLE, the disparity in the observations so far prompts further investigation and the present study was to address this knowledge gap.

With the help of a well-characterized and relatively large *de novo* SLE cohort, and with patients showing variable presentation and divergent responses to immunosuppressive therapy, we have now convincingly demonstrated a reduction in peripheral blood γδT cells in SLE. We also show a negative correlation of this subset of γδT lymphocytes with disease severity and clinical remission following therapy. The negative correlation implies a potentially critical role for the γδT cell compartment in the pathogenesis of SLE and clinical response to therapy. The reason could be that different subsets of γδT cells play different roles in pathogenesis and some subsets may possess more regulatory function while others may be more pro-inflammatory in nature. Hence, further work is necessary to substantiate the underlying mechanism for this observation.

Indeed, the reason for the paucity of peripheral blood γδT cells in active SLE patients remains largely obscure, but we can speculate that it can be related to the tissue distribution of this lymphocyte subset with reference to ongoing pathology and affected organs. The γδT cells are mainly located in relatively tolerogenic environments, in particular in the mucosal surfaces and the associated lymphoid tissue such as the skin, where they may counteract chronic inflammation by supporting innate immunity and thus preventing the festering of small infections. Interestingly, increased numbers of γδT cells have been detected in the non-disease affected skin of SLE patients [17] but also in the damaged skin of chronic cutaneous lupus erythematosus patients [18]. Thus, it is possible that the reduction of peripheral blood γδT cells in SLE relates to extravasations of these cells either to the skin and/or the mucosa. However, the similar number of γδT cells between SLE patients with or without skin lesions [19] suggested that extravasation was not a major influence on blood numbers of these cells. Hence we favor the hypothesis that the low peripheral γδT cell levels *per se* are implicated with the pathogenesis of SLE.

Interestingly and in possible agreement with this notion, γδT cell subsets in SLE patients appear immunologically dysfunctional. An important function of the γδT cell compartment is the secretion of immunomodulatory cytokines that in turn affect disease progression [20–25]. In our study, we observed that the peripheral blood γδT cell subsets secreting cytokines such as IFN-γ, TNF-α and IL-17 are substantially diminished in size in SLE patients. But the levels of serum IL-6, IL-10 and IL-17 were significantly increased consistent with previous research findings [26–28] but not with the level of IFN-γ and TNF-α as compared to the healthy control. Thus IL-17A, IFN-γ and TNF-α levels are inconsistent with the number of γδT cytokine secreting cells. Although IL-17 is a pro-inflammatory cytokine associated with autoimmune phenomena [29], it seems that IL-17-producing γδ T cells are not an important source of this factor. Alternatively, γδT cell compartment may not be associated with hypersecretion of IL-17 in SLE. Previous studies have implicated cytokines such as IL-17A, IFN-γ and TNF-α in the pathogenesis of SLE [30–32], the absence of γδT cells secreting these cytokines may hence influence the progression of SLE by some unknown regulatory mechanism.

The SLEDAI score is generally accepted as a good indicator of disease activity and is associated with the prognosis of SLE and this score was used as an identifier of therapy responders and non-responders using a SLEDAI < = 6.0. In our study, we demonstrated a reduction in γδT cell compartment size cells in onset SLE and gradual recovery after treatment only in the R but not the NR group. On one hand, these dynamics in γδT cell subset size may be due to the effects of drug therapy reflecting its efficacy to disease activity, on the other hand, the NR group was relatively small so they may be subject to spurious variation. Moreover, finding the immunological correlates of the SLEDAI score would be of increased relevance and allow deeper understanding of the effects at work. Here, we provide evidence that the size of TNF-α^+^γδT cells, TNF-α^+^γ9^+^δT cells and IL17^+^CD4^-^CD8^-^γδT cells compartments may be very critical in exploring the incidence and progression of SLE and provide the necessary leads suggesting the need for further investigations exploring how these cells contribute to the induction of the disease.

In summary, we determined the changes in the numbers or compartment size of different subsets of γδT cells before and after therapy in SLE patients. Our data indicate that the number of γδT cells and the subsets in the peripheral blood of SLE patients is substantially lower compared to healthy controls. These numbers recover after treatment and more so in the patients responding well to the therapy. Based on the current observations we suggest that the reduction in TNF-α^+^γδT cells, TNF-α^+^γ9+δT cells and IL17^+^CD4^-^CD8^-^γδT cells might be directly linked to the pathogenesis and progression of SLE, and response to therapy.

## Materials and Methods

### Patients and controls

A total of 22 newly diagnosed SLE patients were recruited from the inpatient service of the First Hospital of Jilin University, Changchun, China between October, 2013 and August, 2014. Patients conforming to the diagnostic criteria as stipulated by the American College of Rheumatology [33] were included in the study. The degree of disease activity was assessed through the SLE disease activity index (SLEDAI) and patients with a score ≥ 6 were considered to be suffering from active disease. Patients suffering from other autoimmune diseases, or had undergone a recent episode of infectious disease, or had received immunosuppressive therapy (including GC therapy) within the last half year before presentation of the current condition (SLE) were excluded from the study. Of the participants included in the present study, 16 SLE patients were available and 6 patients were lost to follow-up after 4 weeks of treatment. And 17 SLE patients were available and 5 patients were lost to follow-up after 12 weeks of treatment. In addition to SLE patients, 14 age and gender-matched healthy controls were recruited from the Physical Examination Center of the First Hospital of Jilin University, Changchun, China for the study. Written informed consent was obtained from all the participants. The study was designed and conducted in accordance with the guidelines of the Declaration of Helsinki and was approved by the human ethics committee of Jilin University. The SLE patients included in this study were treated according to the standard protocols with GC (1 mg/kg/d), HCQ (0.4g/d) (16/22) or intravenous MMF (1.5g/d) (6/22) for the entire study period. All healthy controls were not subjected to medication or other treatment.

### Data collection and clinical evaluation

Demographic and clinical parameters such as age, gender, disease history and current medication regimens were collected from hospital records. Peripheral venous blood was collected before and four and twelve weeks after the onset of treatment and the following parameters were measured–white blood cell (WBC) count, blood platelet count, serum anti-dsDNA, anti-Sm, C-reactive protein (CRP), and complement C3 and C4. Demographic and clinical parameters of the study participants are summarized in Table 1.

### Isolation and stimulation of PBMCs

Peripheral blood mononuclear cells (PBMCs) were isolated by density-gradient centrifugation using Ficoll-Paque Plus (Amersham Biosciences, Little Chalfont, UK) from peripheral venous blood sample (10mL) collected from the study participants. Isolated PBMCs(10^6^ cells/mL) were stimulated using a commercial leukocyte activation cocktail (10μg/mL; Becton Dickinson, USA) in the presence of a protein transport inhibitor containing monensin(3.5μg/ml; Becton Dickinson, USA)while being maintained in RPMI 1640 medium at 37°C in a humidified incubator with 95% air and 5% carbon dioxide for 4 hours. Parallel cultures in which the activation cocktail was not added served as negative control.

### T cell subsets analysis

Stimulated PBMCs were washed and stained with allophycocyanin (APC)-conjugated anti-TCRγδ, phycoerythrin (PE)-conjugated anti-γ9TCR, APC-H7-conjugated anti-CD4, Alexa-Flour488-conjugated anti-CD8, V500-conjugated anti-CD3, PE-Cy7-conjugated anti-CD27 (Becton Dickinson, USA) for 30 min at 4°C. Fluorchrome-conjugated mouse IgG1 isotype antibodies were used as a negative control. Subsequently, 200ulof BD Cytofix/Cytoperm^TM^fixation and permeabilization solution was added to the cells and incubated for 20 min at 4°C. The cells were washed and stained with PE-CF594-conjugated anti-IFN-γ, PerCP-Cy5.5-conjugated anti-TNF-α and Brilliant Violet(BV)421-conjugated anti-IL-17A (Becton Dickinson, USA) to detect intracellular levels of IFN-γ, TNF-α and IL-17A. Stained cells were analyzed using flow cytometry (BD FACSAria^TM^Ⅱ,Becton Dickinson, USA and FlowJo7.6.2 software) to identify the different lymphocytes subsets in the peripheral blood.

### CBA analysis of serum cytokines

The serum concentrations of IFN-γ, TNF-α, IL-2, IL-4, IL-6, IL-10 and IL-17A were determined using CBA [34], according to the manufacturer’s protocol (BD Biosciences) with minor modifications using a flow cytometer (BD FACSAria^TM^ II, Becton Dickinson, USA). The concentrations of serum cytokines were quantified using the Cell Quest Pro according to routine procedures and CBA software (Becton Dickinson) on a FACSAria II.

### Statistical Analysis

Statistical tests such as Fisher exact test and Kruskal-Wallis H non-parametric test was used to analyze differential observation between the groups. Data are expressed as mean ±SEM unless otherwise specified. A two-side *P* value of < 0.05 was considered to be statistically significant. All statistical analyses were performed using the SPSS 19.0 software (SPSS, Inc, Chicago, IL, USA).

## Supporting Information

S1 Database The database includes all the number of γδ T cell subsets and cytokines measured in the research. (XLSX)
